# Reformed child and adolescent mental health services in a devolved healthcare system: a mixed-methods case study of an implementation site

**DOI:** 10.3389/frhs.2023.1112544

**Published:** 2023-05-05

**Authors:** Emily Banwell, Neil Humphrey, Pamela Qualter

**Affiliations:** Manchester Institute of Education, University of Manchester, Manchester, United Kingdom

**Keywords:** mental health services, child and adolescent mental health, reformed health services, implementation science, programme evaluation

## Abstract

**Background:**

Efforts are being made to reform and reconceptualise children and young people's (CYP) mental health services. This is in response to a rapid increase in mental health difficulties in this population, and the shortcomings of current service provision. The present study seeks to comprehensively evaluate the local implementation of the THRIVE Framework for System Change in Greater Manchester, UK (GM i-THRIVE) from 2018 to 2021. The framework was designed to change the way mental health is perceived, and subsequently how support is allocated. The current study focusses on the implementation of the framework's principles into CYP mental health support in the region.

**Methods:**

The study comprised three methodological components, beginning with examination of the GM i-THRIVE implementation plan and self-assessment questionnaire measure using the Quality Implementation Tool. This was to provide a wider backdrop of implementation method adequacy to the rest of the study's findings. Subsequently, evaluation measures completed by professionals across Greater Manchester were examined to establish implementation progress, before corroborating key items from this measure with thematically analysed interview data from six CYP (13–22 years) who recently received mental health support in the region. Levels of agreement between staff and CYP were examined.

**Results:**

GM i-THRIVE's implementation plan and self-assessment measure were respectively deemed a strong guiding foundation, and a suitable way of evaluating implementation progress. Every principle within the self-assessment measure demonstrated closer alignment with the THRIVE Framework as time progressed. Two themes were developed from the qualitative interview data, each overarching four subthemes: (1) *Qualities of the service:* information and decision sharing; communication and continuity; needs-based support; compassion and trust, and (2) *The mental health journey:* beginnings; endings; waiting; satisfaction with support. A good level of agreement between CYP testimony and staff progress reports was found.

**Conclusions:**

Findings suggested that the experiences of the CYP in the sample, who were interviewed in the spring to summer period of 2022, were overwhelmingly positive. The rich insights into mental health support offered by the young participants lead us to recommend continued qualitative research with service-users as GM i-THRIVE's embedding period continues, with focus on representing a wide range of experiences in future research samples. Methodological limitations were explored, including the extent to which true cross-references could be made between professional and CYP accounts.

## 1. Introduction

### 1.1. Background

The prevalence of mental health difficulties in children and young people (CYP) is increasing year on year (1). The peak age of onset for all mental health conditions is 14.5 years (2), with 75% of all mental health conditions appearing before a person reaches their mid-twenties (3). Accordingly, efforts to ameliorate the impact of these difficulties as early as possible should be policy priority, as is, consequently, the meticulous evaluation of these efforts. The present study provides an in-depth mixed-methods evaluation of how successfully the THRIVE Framework for System Change (4) is being implemented in Greater Manchester, United Kingdom.

**Figure 1:**
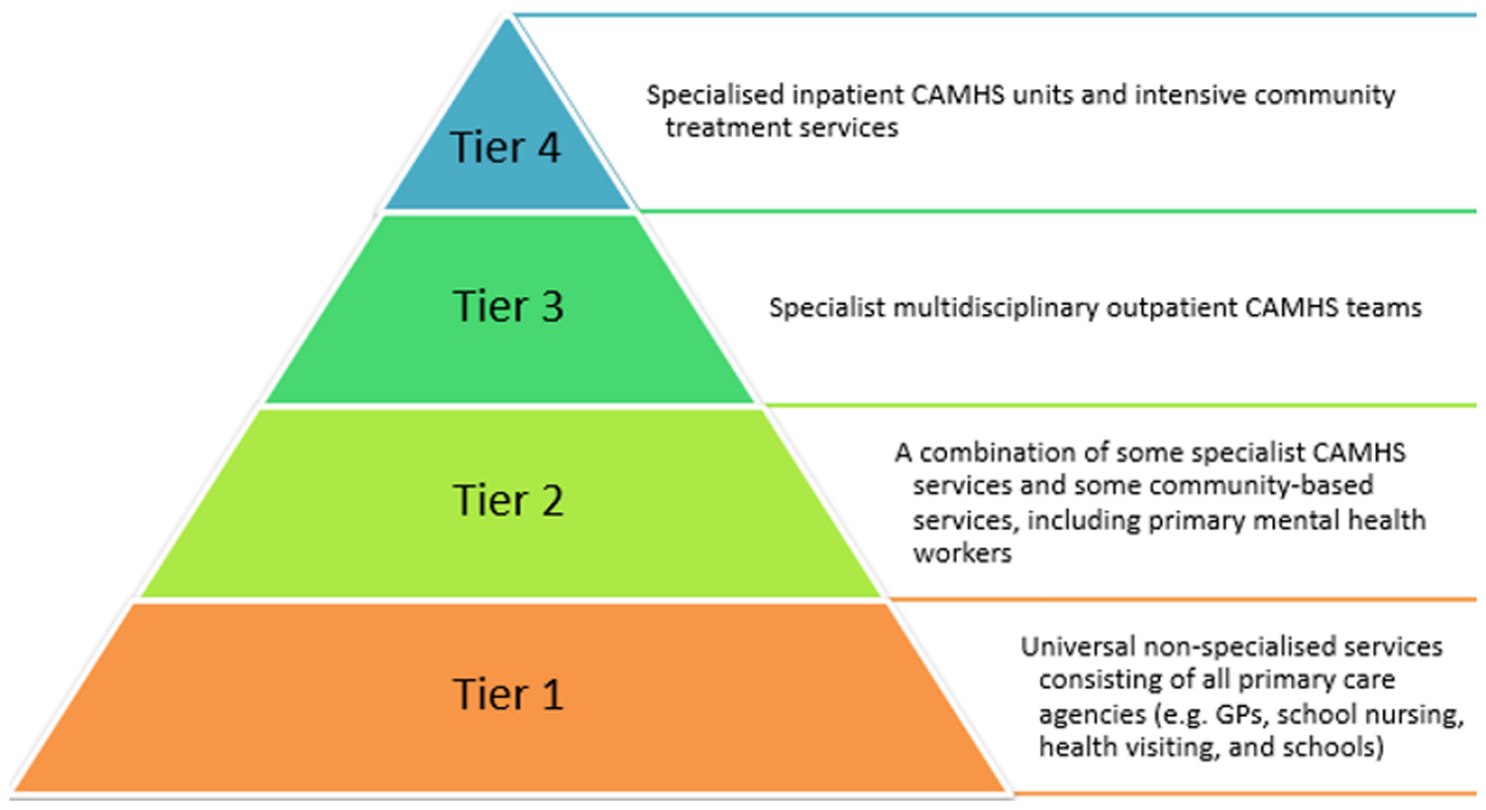
CAMHS tiered model of service conceptualisation.

To fully understand what the implementation of THRIVE, a national reconceptualisation of CYP mental health and service provision in England, hopes to achieve, we must first explore the various ways that previous provision of child and adolescent mental health services [known by the acronym of CAMHS when this refers to services provided by the National Health Service (NHS) in the UK] fell short of providing adequate support. The key inadequacy was that appropriate specialist support was often tremendously difficult for CYP to access. For example, in 2018, only 25% of CYP with a diagnosable condition managed to utilise specialist CAMHS services in England (5, 6). Reduced government spending allocation to mental healthcare provision (7) including to CAMHS (8), substantial waiting times (9, 10), and high referral rejection rates (11) may contribute towards our understanding of why, despite the rise in demand for these services (1), so many remain unseen by specialist mental health professionals. In addition to those likely explanations, the rigid nature of how mental health services were conceptualised by CAMHS was, by its nature, prohibitive to CYP receiving appropriate and timely support. The tiered model (see Figure 1) that has dominated CAMHS provision since its 1995 inception (12) meant that accessing specialist support required contact with a myriad number of professionals across the tiers before finally receiving appropriate care (5, 13). The model has been criticised for unnecessarily compartmentalising services and their provision (14); a reification that has resulted in many being unable to receive support, or “falling between the gaps”, if they do not perfectly fulfil the criteria pertaining to a certain tier (5, 10).

### 1.2. The THRIVE framework for system change

The THRIVE Framework, adopted by more than 70 localities in England to date, aims to improve access to mental health services for CYP in many important ways. One of these is to disseminate the idea that CYP mental health is “everybody”s business” (15): that responsibility should not, and indeed *does* not, belong solely to medicalised services that are provided by the NHS. Allied professionals, of which teachers are a prime example, are essentially a “front line” source of mental health support for CYP (16). These trusted adults are often relied upon because of the widespread inability to access CAMHS services outlined above, but also because not all wellbeing and mental health concerns require intervention from a medicalised service. A negative emotional reaction to, for example, a bereavement or a parental separation, is healthy and expected, yet appropriate support is still required to prevent the disturbance from persisting. THRIVE recognises, therefore, that anyone who comes into professional contact with CYP should be well-placed to provide such support or guidance. However, many allied professionals currently feel ill-prepared to assist to the level that they wish they were able (16). Thus, THRIVE is training a diverse range of these professionals so that they can provide a more inclusive, seamless, and accessible support network. This should, ideally, lead to a scenario where is never a “wrong door” in which to turn, owing to a widespread and consistent standard of support and signposting (5).

**Figure 2:**
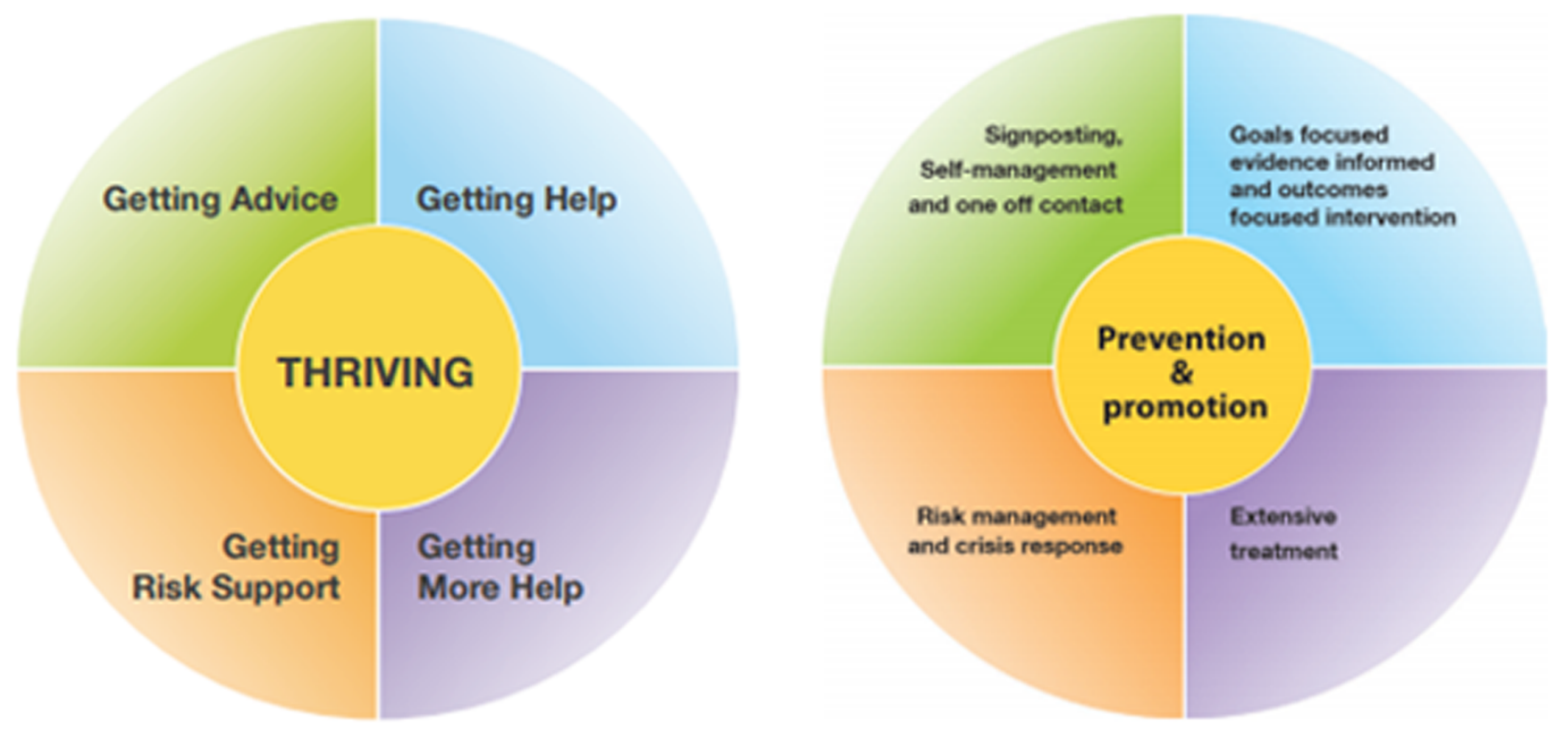
The THRIVE model. Left: THRIVE's five needs-based groupings. Right: the support that CYP mental health needs can benefit from under each grouping (10).

Preventative mental health support for CYP, by way of deescalating concerns before they exacerbate, is a key step towards breaking the commonly seen associations between poor mental health in early life and detrimental outcomes in adulthood (17, 18). The fact that medicalised support is at the heart of the tiered model means that support can only be given when a problem has escalated to a certain point. THRIVE, on the other hand, advocates a needs-based approach, whereby support is provided based upon present requirement, irrespective of previous diagnoses or service use (4). This means that every young person is accounted for by one of the five needs-based groupings of the THRIVE model (Figure 2). It is acknowledged that everyone can benefit from some form of support, depending upon which grouping their needs fall under at any given time.

By offering diversified options for receiving mental health support, and ensuring that more CYP can receive it from any professional they meet in their day-to-day life, the implementation of THRIVE should result in reduced waiting times for specialised CAMHS services and an availability of alternative resources whilst a wait is in progress. An ethos of open communication will mean than decisions are undertaken using a cross-sector approach, eradicating the “silo mentality” that is regarded as a prominent issue across the wider NHS (14, 19) but notably within CAMHS, where a lack of accountability for certain elements of care is a common feature (5). The involvement of CYP and their families at every step of this decision-making also features in THRIVE-aligned support (4) resulting in care that is substantially more tailored towards each young person.

Since 2018, Greater Manchester's ten locality boroughs (Bolton, Bury, Manchester, Oldham, Rochdale, Salford, Stockport, Tameside, Trafford, and Wigan) have gradually aligned their CYP mental health services to the THRIVE Framework. This alignment process, known locally as GM i-THRIVE, is just one element of devolved health and social care resulting from a 2016 deal between the Greater Manchester Health and Social Care Partnership (GMHSCP) and the UK government. GMHSCP can now decide how services are funded at a local level, meaning that spending can be allocated appropriately to the 2.8 million residents of Greater Manchester. An initial implementation period of four years (2018–22) was given to introduce, implement, and normalise the Framework within all sectors that provide mental health support for CYP. It is therefore crucial that this formal implementation phase provides the strongest possible foundation for the ongoing success of the programme. To do this, a careful, iterative process of planning, implementing, and monitoring (20) is essential, with consideration given to the impact of the COVID-19 pandemic on both service provision, and the delivery and evaluation of the programme.

### 1.3. The present study

By combining a variety of methodological approaches, the present study aimed to evaluate GM i-THRIVE's implementation progress to date. At the time of research, the four-year initial implementation period (2018–22) was ending, and a short “embedding” phase, in which implementation efforts are continuing, was beginning. This meant that sufficient information, data, and informed testimonies were available with which to conduct a comprehensive evaluation. The components of the present study and their objectives will now be outlined in turn.

First, through qualitative document analysis, we assessed the adequacy of GM i-THRIVE's own implementation plan using the Quality Implementation Tool (QIT) (21). The plan documented the action steps, spanning from conceptualization through to sustainability, that guide the implementation process (see Supplementary Materials*).* Second, we established whether the aims of GM i-THRIVE were met, by analysing ratings of progress, self-reported by professionals working across Greater Manchester. Finally, interviews were conducted with CYP who were recently in receipt of support from THRIVE-aligned mental health services in Greater Manchester. This service-user data was compared to the implementation progress reported by the localities. Whilst localities might report a certain level of implementation progress, if CYP in Greater Manchester do not describe experiences that evidence THRIVE-aligned care, such reports would mean very little. Young people's hopes and expectations of the outcomes of mental health care often considerably differ from those of the adults involved in their support. Research has revealed that parents and their children have conflicting ideas of what ideal CAMHS provision would look like (22). Differences also exist between CYP, parents, and therapists in terms of what mental health improvement, and desired outcomes of support, look like (22, 23). This lack of consensus can have a detrimental impact on CYP engagement with services, leading to disconnection within the therapeutic relationship, and ultimately, poorer support outcomes (23). These studies suggest that young people's insights provide a valuable source of information, which is often underutilised. Within our study, it followed that their experiences, opinions, and indeed disagreements, could and should be meaningfully compared with localities’ reports of progress to form a comprehensive evaluation. To summarise the above components, the key research questions for the present study were as follows:

1. Do GM i-THRIVE's overarching implementation plan, and self-assessment evaluation system, contain the components deemed necessary (21) for successful implementation and evaluation of an intervention?
2. Do the localities within Greater Manchester report a general shift towards aligning their practices with the THRIVE Framework within the four-year initial implementation period?
3. Do the experiences of CYP receiving mental health care in Greater Manchester align with the implementation progress reported by localities?

## 2. Method

### 2.1. Reporting guidelines

The production of this article adhered to the Standards for Reporting Qualitative Research (SRQR) (24). In addition, the principles of reflexive thematic analysis (25, 26) were used to guide the reporting and analysis of the qualitative data.

### 2.2. Researcher context

The authors were externally commissioned by GMHSCP to evaluate GM i-THRIVE. As employees of the University of Manchester rather than GMHSCP, the analyses and conclusions drawn in this study were unlikely to be biased by vested interest. However, the first author has been continually immersed in the working environment of GM i-THRIVE as part of this work (e.g., as an attendee of regular meetings with key leaders and stakeholders). As a result, impressions gained during these meetings may have influenced the analysis of the present study's qualitative data (27). The knowledge of salient issues and working practices may have guided theme production, even at a subconscious level. Despite this, the immersive experience provided an in-depth knowledge of the people, practices, and systems of GM i-THRIVE that was undoubtedly advantageous. It provided understanding and empathy to the analysis Considering this situation in tandem with the principles of reflexive thematic analysis (25), conclusions drawn can only ever reflect the author's interpretation of the qualitative data. Whilst this subjectivity should certainly be considered alongside this study's findings, it should be viewed as a tool that sculpts the analysis rather than as a threat to credibility (25).

### 2.3. Setting

The implementation site of Greater Manchester, which was home to 898,000 under-25s in 2019 (28), is an ethnically and socially diverse city-region in the north-west of England. It contains a mix of high-density urban areas, suburbs, and rural locations within its 493 square mile boundaries. CYP living in Greater Manchester are more likely to live in poverty and have poorer overall health outcomes than the average in the UK (28). The city-region comprises ten metropolitan boroughs (Bolton, Bury, Manchester, Oldham, Rochdale, Salford, Stockport, Tameside, Trafford, and Wigan), all of which have a dedicated team responsible for coordinating the implementation of GM i-THRIVE across specialist NHS CYP mental health services, and other local service providers.

### 2.4. Design

The present study was a mixed-methods case study of GM i-THRIVE. It combined qualitative and quantitative document analyses with semi-structured qualitative interviews. This triangulation enabled the generation of comprehensive meta-inferences, pertaining not only to implementation progress, but also to how successfully it was planned and measured. Acknowledging one's reasons for adopting a mixed methodological approach is an important part of the rationale behind any evaluation design (29). One of our broad research aims was to counteract the potential bias in localities' self-reports of progress with qualitative accounts from CYP. This served to strengthen the validity of our inferences, as per “triangulation” in its most classic sense (29). However, the discovery of paradox between the various testimonies in the present study was a key driver of interest. The potential conclusions drawn from discrepancies can indeed be just insightful as consistencies in research of this nature.

We deduced that the most appropriate way to approach the evaluation of GM i-THRIVE was through a pragmatic epistemological lens. The assortment of methods used in the present study were chosen purely for their ability to meet each research aim. The pragmatic notion that knowledge of the inner workings of organisations can be generated through the conflation of participant accounts with the empirically measurable (30) meshes extremely well with our study aims. Beyond this, a deeper degree of reflection on the formulation of knowledge was simply not needed for an evaluation of this kind (31).

### 2.5. Participants

Eligibility for the qualitative element of the study required participants to have received mental health or wellbeing support since September 2018: the start of GM i-THRIVE's implementation period. This support must have come from a site or service within Greater Manchester that was active in the process of aligning their practices to the THRIVE Framework. Participants needed to be aged 13 or over, and to have been in receipt of support from a CYP mental health service at any time from 2018 onwards, regardless of when their support began. We did not set a strict upper age limit, given that some local services focus only on those under 18, whereas some stretch to 21 years old. 13 was deemed a suitable lower cut-off age at which participants could properly assent to and engage with the research. Participants were identified on the basis that they were either former users of a service, or they were in the final stages of receiving support. These criteria ensured that the mental health of participants was sufficiently stable to both assent and take part. A gatekeeper within the GM i-THRIVE implementing team identified participants through their support providers on an opportunistic basis. They were approached based on the providers' perception of them as able and willing to participate in an interview, with a third party, about their experiences with support. Participants were given the option of having another person present to provide emotional support.

### 2.6. Ethical considerations

Ethical approval was received (reference number: 2021-11033-18945) from the University of Manchester's research ethics committee (UREC). All participants (and their parents if under 16) were provided with age-appropriate participant information sheets, detailing the nature of the study and their potential contribution. Written consent was obtained from participants who were over 16 (in the UK, this is the age that a person is thought able to independently provide full consent to research participation), and from the parents of the 13- to 15-year-olds. 13- to 15-year-olds gave written assent to take part, confirming that they understood the study and how their data would be used. Through a process of reinstating information and rights, and being attuned to our participants' responses and body language, consent, or dissent, was obtained continuously and reflexively as per a recent reframing of research consent (32).

### 2.7. Data collection

#### 2.7.1. Secondary data for document analyses

GM i-THRIVE provided a copy of their implementation plan, which comprised five overarching stages: set-up; engagement, understanding, and planning; building capacity; implementation; and embedding and sustaining. Each stage contained several granular items that were to be completed during the implementation process. A copy of this implementation plan can be found in the Supplementary Materials. Self-assessment matrices were completed annually by each Greater Manchester locality. These provided a report of perceived alignment over time to the THRIVE model. At the beginning of implementation in 2018, completions of the matrix generated a baseline “snapshot” of practices, whilst subsequent completions indicated the success of individual localities' transformation strategies. The matrix outlines 22 underlying principles of the THRIVE Framework that are divided into three categories: micro (considerations for individual CYP and professionals), meso (community-level considerations), and macro (larger-scale considerations for the wider population). The matrix then allows the locality to rate their progress from 1 (“some way to go to achieving THRIVE-like practice”) to 4 (“practice is very THRIVE-like”). Detailed commentaries were provided alongside each principle to help guide selection. Completed matrices from 2018 to 21 were provided to the authors for secondary analysis. A list of the matrix's principles can be found in Table 1.

**Table 1:** Principles of the GM i-THRIVE self-assessment matrix.

Principle of GM i-THRIVE self-assessment matrix	Description
MACRO PRINCIPLE 1:	A locality's mental health policy is interagency.
MACRO PRINCIPLE 2:	All agencies are involved in commissioning care (education, health, social care, third sector)*
MACRO PRINCIPLE 3:	Contracting of services, and the performance management of these, is informed by quality improvement information
MACRO PRINCIPLE 4:	Use of population level preference data is used to support commissioning decisions.
MACRO PRINCIPLE 5:	Services working closely together such that service users experience integration of care positively*
MESO PRINCIPLE 1:	A comprehensive network of community providers is in place
MESO PRINCIPLE 2:	Quality Improvement (QI) data used to inform decisions, and this involves multiagency consideration of the data
MESO PRINCIPLE 3A:	Help is delivered using the conceptual framework of five needs based groups
MESO PRINCIPLE 3B:	As above, but based on results of staff survey about whether they think care is delivered in this way (what % of staff)*
MESO PRINCIPLE 4:	There is a focus on strengths and family resources wherever possible
MESO PRINCIPLE 5:	Evidence based practice is available and aligned to need using the 19 sub categories of needs as set out in the payment systems work
MICRO PRINCIPLE 1A:	Shared Decision Making (SDM) at the heart of all decisions (based on perceived implementation extent)*
MICRO PRINCIPLE 1B:	As above, but based on scores on CollaboRATE (what % of CYP given the chance to rate their experience of SDM)
MICRO PRINCIPLE 2:	People (staff, CYP and families) are clear about which needs based group they are working within for any one person at any one time and this explicit to all*
MICRO PRINCIPLE 3A:	People (staff, CYP and families) are clear about parameters for help and reasons for ending (staff survey)*
MICRO PRINCIPLE 3B:	As above, but based on % of cases with reasons for ending included in proforma and endings discussed with CYP at start
MICRO PRINCIPLE 3C:	As above but based on if staff had training on this/recognise it as an important part of therapy*
MICRO PRINCIPLE 4:	Outcome data is used to inform individual practice with the purpose of improving quality
MICRO PRINCIPLE 5A:	Any intervention would involve explicit agreement from the beginning about the outcome being worked towards and the likely timeframe. There would be a plan for what happens if it is not achieved. (% that are managed in recommended timeframe)*
MICRO PRINCIPLE 5B:	As above, but notes include info on goals/outcomes discussion with CYP*
MICRO PRINCIPLE 6:	The most experienced practitioners inform advice and signposting
MICRO PRINCIPLE 7:	THRIVE plans are used to help those managing risk (Case audit: % of CYP in the “Getting Risk Support” needs based group have a THRIVE plan documented and up to date)

**Figure 3:**
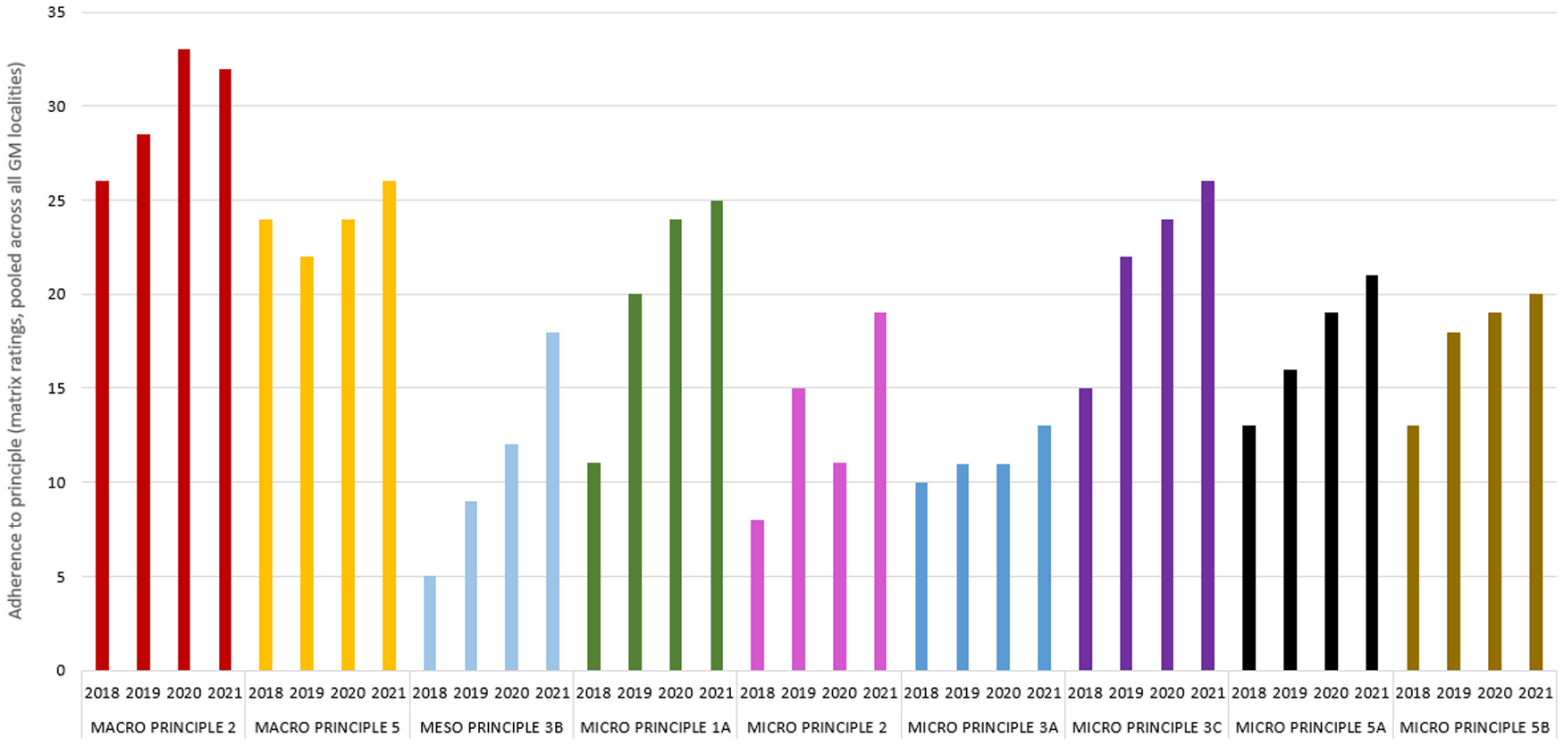
Reported adherence to nine principles of the self-assessment matrix. Out of the 22 total principles, these nine were selected for analysis based on their closer relevance to the aims of the study. Details of principles can be found in Table 1.

* Those selected for presentation in Figure 3, and for comparison with the qualitative themes.

#### 2.7.2. Interview data

Due to social distancing restrictions enforced in response to the COVID-19 pandemic, interviews were conducted by the first author using secure online video conferencing software. Semi-structured interviews were used to explore participants' experiences of receiving recent support for their mental health. The interview schedule was designed to ascertain the extent to which the aims of THRIVE were reflected in the participants' experiences of support or care. The schedule consisted of 10 broad questions, overarching several prompts and sub-questions (see Supplementary Materials).

### 2.8. Data analysis

#### 2.8.1. Document analyses

For the first step of the document analysis, GM i-THRIVE's implementation plan and the blank self-assessment matrix were checked, together, for the presence of each of the 28 action steps of the QIT (21) (see Table 2). The QIT, a practical translation of the Quality Implementation Framework (20) comprises check-list style action steps that provide a blueprint for high-quality implementation of evidence-based interventions (see Table 2). The QIT is a flexible tool that can be used in all stages of the implementation process, from iteratively guiding design and implementation, through to reflective evaluation (21). The decision to check the two documents together owed to the fact that the 28 steps are divided into six overarching components of implementation quality: the first five dealing with the set-up of the intervention, from developing teams, to training, and component 6 focussing solely on evaluating the intervention once it has begun. We thought it appropriate to evaluate the implementation plan using components 1–5, and the self-assessment matrix with component 6. These findings were tabulated (Table 2). Completed self-assessment matrices from 2018 to 21 were used to produce a bar chart to show reported adherence to each principle across the initial implementation period of 2018–2021 (Figure 3). As our interest was in overall adherence across Greater Manchester, localities' responses were pooled for this analysis component. Each locality's individual scores for each principle were therefore summed producing an overall score. Of the 22 principles, only nine, those related to staff opinions of CYP experiences, were selected for presentation in Figure 3, and to compare with the qualitative data, given their relevance to the study's aims (see Table 1). However, line graph visualisations were produced for all 22 principles to show their reported change over time. These can be found in the Supplementary Materials Section.

**Table 2:** The components and action steps of the QIT, and whether they were evidenced in GM i-THRIVE's implementation plan and self-assessment matrix.

Component of QIT	Action step	Stage of GM i-THRIVE implementation that action step relates to	Document checked for action step	Was this step present in the document?	Examples from plan/comments
**1. Develop an implementation team**					
	1.1 Decide on structure of team overseeing implementation (e.g., steering committee, advisory board, community coalition, workgroups, etc.)	Implementation set-up	GM i-THRIVE implementation plan	Yes	Point 0.4*: Undertake stakeholder mapping
	1.2 Identify an implementation team leader	Implementation set-up	GM i-THRIVE implementation plan	Yes	Point 0.2*: Have a named lead for implementing THRIVE
	1.3 Identify and recruit content area specialists as team members	Implementation set-up	GM i-THRIVE implementation plan	Yes	Point* 0.6: Multi-agency working group established
	1.4 Identify and recruit other agencies and/or community members such as family members, youth, clergy, and business leaders as team members	Implementation set-up	GM i-THRIVE implementation plan	Yes	Point 0.3*: Set up multi-agency Programme Board [include senior leadership from CCG, health provider(s), local authority, education, third sector]
	1.5 Assign team members roles, processes, and responsibilities	Implementation set-up	GM i-THRIVE implementation plan	Unclear	Although not explicitly mentioned, this process is likely captured in points 0.1–0.10*, and 2.0–2.12*
**2. Foster supportive organizational/communitywide climate and conditions**					
	2.1 Identify and foster a relationship with a champion for the innovation	Implementation set-up	GM i-THRIVE implementation plan	Yes	Point 2.7*: Identification and creation of local champions and implementation leads
	2.2 Communicate the perceived need for the innovation within the organization/community	Implementation set-up	GM i-THRIVE implementation plan	Yes	Point 1.8*: Service performance review (including population need, demand, flow, experience of service, participation levels, clinical outcomes, efficiency, current shared decision-making practice etc)
	2.3 Communicate the perceived benefit of the innovation within the organization/community	Implementation set-up	GM i-THRIVE implementation plan	Yes	Point 1.1*: Key messaging for i-THRIVE project established—goals, aspirations, local context
	2.4 Establish practices that counterbalance stakeholder resistance to change	Implementation set-up	GM i-THRIVE implementation plan	Unclear	Not explicitly mentioned, but Point 0.1*: Establish cross sector approval to proceed with i-THRIVE, which suggests commitment before proceeding
	2.5 Create policies that enhance accountability	Implementation set-up	GM i-THRIVE implementation plan	Unclear	Not explicitly mentioned, but training implementation monitored: Point 2.12*: Review of workforce development delivery and plans for ongoing work
	2.6 Create policies that foster shared decision-making and effective communication	Implementation set-up	GM i-THRIVE implementation plan	Yes	Point 0.8*: Establish communications functions (contact databases, shared folders, website)
	2.7 Ensure that the program has adequate administrative support	Implementation set-up	GM i-THRIVE implementation plan	No	Not mentioned
**3. Develop an implementation plan**					
	3.1 List tasks required for implementation	Implementation set-up	GM i-THRIVE implementation plan	Yes	Point 1.13*: Prioritisation and gap analysis workshop
	3.2 Establish a timeline for implementation tasks	Implementation set-up	GM i-THRIVE implementation plan	Yes	Point 1.16*: Finalise implementation plan
	3.3 Assign implementation tasks to specific stakeholders	Implementation set-up	GM i-THRIVE implementation plan	Yes	Point 3.2*: Detailed implementation planning finalised with lead for each project identified
**4. Receive training and technical assistance (TA)**					
	4.1 Determine specific needs for training and/or TA	Implementation set-up	GM i-THRIVE implementation plan	Yes	Point 2.1*: Review of staff skills for THRIVE-like working
	4.2 Identify and foster relationship with a trainer(s) and/or TA provider(s)	Implementation set-up	GM i-THRIVE implementation plan	No	Not mentioned
	4.3 Ensure that trainer(s) and/or TA provider(s) have sufficient knowledge about the organization/community's needs and resources	Implementation set-up	GM i-THRIVE implementation plan	No	Not mentioned
	4.4 Ensure that trainer(s) and/or TA provider(s) have sufficient knowledge about the organization/community's goals and objectives	Implementation set-up	GM i-THRIVE implementation plan	No	Not mentioned
	4.5 Work with TA providers to implement the innovation	Implementation set-up	GM i-THRIVE implementation plan	No	Not mentioned
**5. Practitioner–developer collaboration in implementation**					
	5.1 Collaborate with expert developers (e.g., researchers) about factors impacting quality of implementation in the organization/community	Implementation set-up	GM i-THRIVE implementation plan	Unclear	Touched upon in point 1.8*: Service performance review (including population need, demand, flow, experience of service, participation levels, clinical outcomes, efficiency, current shared decision-making practice etc)
	5.2 Engage in problem solving	Implementation set-up	GM i-THRIVE implementation plan	No	Not mentioned
**6. Evaluate the effectiveness of the implementation**					
	6.1 Measure fidelity of implementation (i.e., adherence, integrity)	Implementation evaluation	Self-assessment matrix	Yes	Micro principle 7: THRIVE plans are used to help those managing risk
	6.2 Measure dosage of the innovation—how much of the innovation was actually delivered	Implementation evaluation	Self-assessment matrix	Yes	Meso principle 3A: Help is delivered using the conceptual framework of five needs-based groups—measure asks how many groups have been implemented
	6.3 Measure quality of the innovation's delivery—qualitative aspects of program delivery (e.g., implementer enthusiasm, leader preparedness, global estimates of session effectiveness, leader attitudes towards the innovation)	Implementation evaluation	Self-assessment matrix	Yes	Micro principle 4: Outcome data is used to inform individual practice with the purpose of improving quality
	6.4 Measure participant responsiveness to the implementation process—degree to which participants are engaged in the activities and content of the innovation	Implementation evaluation	Self-assessment matrix	Yes	Micro principle 1: Shared Decision Making (SDM) at the heart of all decisions
	6.5 Measure degree of program differentiation—extent to which the targeted innovation differs from other innovations in the organization/community	Implementation evaluation	Self-assessment matrix	Yes	Micro principle 2: People (staff, CYP and families) are clear about which needs based group they are working within for any one person at any one time and this explicit to all
	6.6 Measure program reach—extent to which the innovation is delivered to the people it was designed to reach	Implementation evaluation	Self-assessment matrix	Yes	Micro principle 3B: People (staff, CYP and families) are clear about parameters for help and reasons for ending—measured by % of cases with reasons for ending included in case notes
	6.7 Document all adaptations that are made to the innovation—extent to which adjustments were made to the original innovation or program in order to fit the host setting's needs, resources, preferences, or other important characteristics	Implementation evaluation	Self-assessment matrix	No	Not mentioned

* Points in this column refer to those in the GM i-THRIVE implementation plan (see Supplementary Materials).

#### 2.8.2. Qualitative interview data

Participant recruitment and interviews took place between April and July 2022. Interviews were securely recorded *via* the video conferencing software, and the automatically generated transcripts were checked manually for accuracy by the first author. Data were analysed following Braun & Clarke's guidelines for reflexive thematic analysis (25, 26). The flexible application and broad epistemological compatibility of this approach (25) made it a suitable way of exploring ourresearch aims. The simple yet rich organisational data summary that the method lends itself to when analysis is complete (26) was also appealing given that meta-inferences were to be drawn. Thematic analysis allows both inductive and deductive code and theme identification methods (26). Given that the purpose of interviewing CYP was to establish whether their reported experiences matched locality-reported progress, this aim acted as a key driver of the analytic strategy. Initially, therefore, a deductive strategy was used to code the data. For this, a list of provisional codes was generated based upon the principles of the matrix (Table 1). However, new codes were generated when other notable features were identified in the transcripts, adding an inductive element to the analysis. When all codes were developed, they were renamed as appropriate more suitably fit the data.. Codes were then grouped into semantic themes, which were tested and refined reflexively (25) with each transcript, and with the entire data set. A dynamic thematic map was developed to assist this non-linear process. Final themes were then defined, and named in a way that any inconsistencies in CYP's testimonies were still suitably covered by the theme title. Whilst the study's aim was to compare the themes and their content to matrix data, these final themes were not forced to match the principles, rather, they were named to encapsulate their interpretative nature (25), and encapsulate the experiences of the participants as appropriately as possible. The process of naming and re-naming themes continued into the write-up stage of the analysis.

The thematic analysis was completed prior to the document analyses, to avoid the risk of unintended bias that may have come from the results of the matrices. Researcher subjectivity is not seen as problematic in thematic analysis (25, 33). Rather, it should be seen as a resource for reflexive data analysis and as an asset to knowledge production (34). Pursuing researcher consensus, given that interpretation, rather than objective “accuracy”, is the goal of thematic analysis, is also discouraged (34). However, the broad processes of theme generation and mixed-methodological cross-referencing were nonetheless sense-checked by the second and third authors (34). This was to ensure that the themes appeared to represent the data logically (35) and that the interpretation was as rich as possible (34).

#### 2.8.3. Meta-inferences

Staff and CYP accounts were considered within the boundaries of the quality of the implementation plan, and of the self-assessment matrix, as determined by the QIT (21). The self-assessment matrix data and the qualitative interview data were analysed together in a simultaneous bidirectional manner (36). This means that both strands were considered as equally important when overarching conclusions were drawn (37). When all analyses were complete, the themes and their extracts were compared, one by one, to the principles of the self-assessment matrix, to cross-reference accounts of progress where possible. A theme was deemed to “match” a principle if the broad topics covered within the participant extracts were similar. Owing to the nature of some themes, a match was occasionally established with more than one principle. For example, in the first subtheme “information and decision sharing”, participants discussed the sharing of decisions and the discussion of outcomes. Micro principles 1A and 5B, which cover joint decisions and discussions, including those made around support goals (see Table 1), were both deemed to match this. Under each relevant subtheme, the extent to which CYP accounts substantiate staff accounts from the conceptually closest matrix principle is denoted as high, moderate, or low. This was done by examining the experiences reported in each theme to establish whether these were positive, negative, or mixed. Returning, again, to the subtheme “information and decision sharing”, staff reported modest yet gradually improving adherence to micro principles 1A and 5B, which corresponds with the diverse testimonies relating to them. Please note that not all subthemes suitably matched a principle. Given that uncovering paradox was a key motivation of mixing methods in the present study, consistencies *and* discrepancies between the matrix and the themes were given equal attention and status in this final part of the analysis (29). This equal interest in discrepancy provides another explanation as to why it was not important for themes to perfectly match the matrix principles. This is also the reason why not all qualitative themes below correspond to a principle. The meta-inferences were primarily made by the first author, with additional input and “sense-checking” provided by the second and third authors.

## 3. Findings

The GM i-THRIVE implementation plan and self-assessment matrix fulfilled 62.1% of the criteria for quality implementation outlined in the QIT (21) (see Table 2). Of the 29 action steps outlined in the tool, 18 (62.1%) of these were explicitly evidenced in the plan, and 7 (24.1%) were not mentioned. It was not clear whether the remaining 4 (13.8%) steps were covered by the plan: steps were assessed as “unclear” if their fit with the plan was ambiguous.

Figure 3 shows Greater Manchester's self-reported adherence to the principles in Table 1 from 2018 to 2021.

6 participants were recruited by the gatekeeper and were interviewed between April and June 2022. Interviews ranged from between 15 and 30 min long, depending on the level of detail that participants expanded upon. Their participant numbers (which correspond to transcript extracts provided in the thematic analysis) and ages can be found in Table 3.

**Table 3:** Participant numbers and their ages.

Participant number	Age
1	18
2	16
3	13
4	14
5	20
6	22

**Figure 4:**
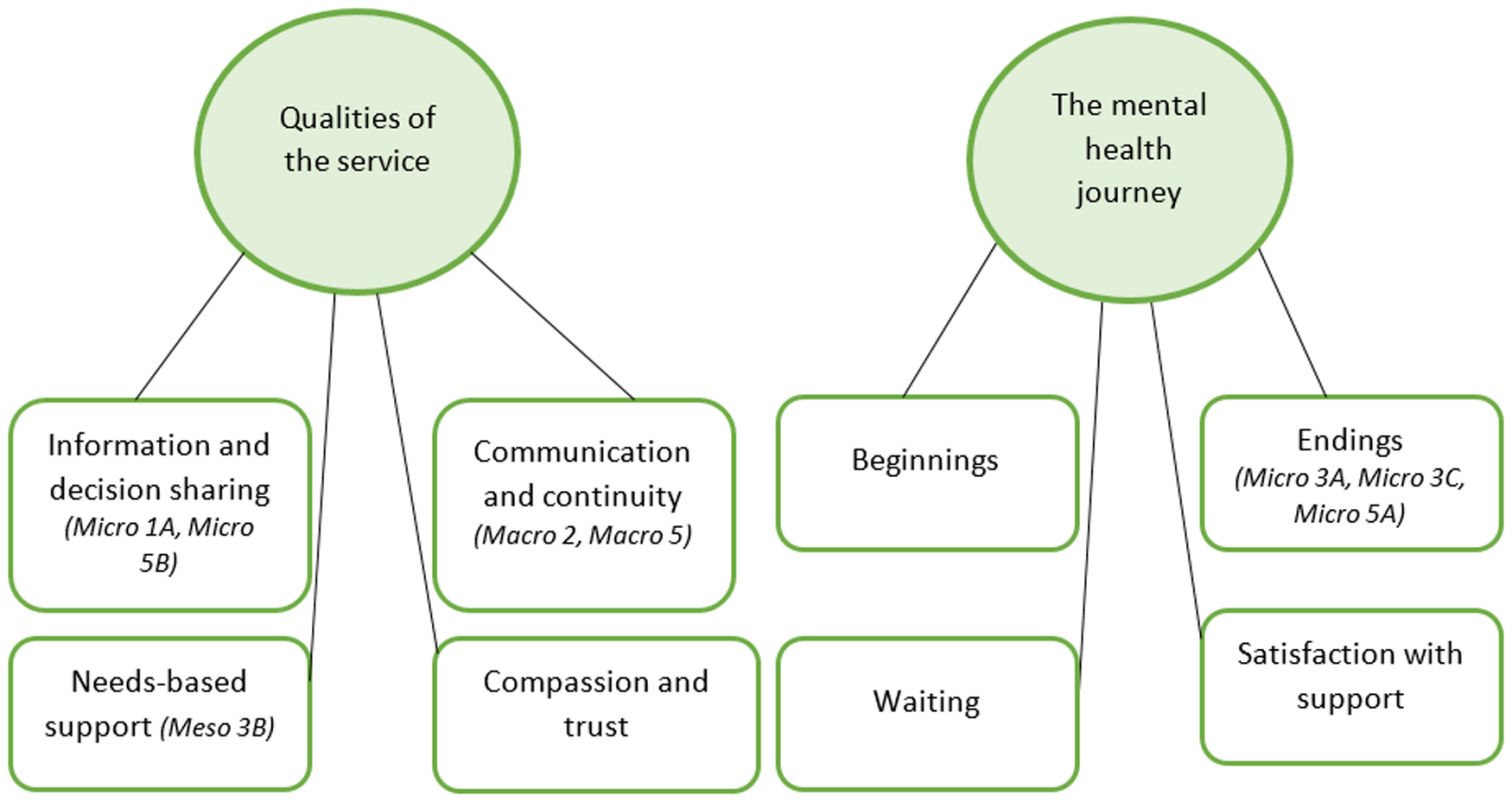
Map of the final themes and subthemes that were created through thematic analysis.

Two themes were developed through reflexive thematic analysis, each of which overarched four subthemes (see Figure 4). These are explored in turn, using illustrative examples from the transcripts. Links to the self-assessment matrix, and the findings outlined in Figure 3, are made after each subtheme where appropriate. Detailed explanations of how each level of agreement was established can be found within the discussion section.

### 3.1. Qualities of the service

This theme included descriptions of what participants felt that the service offered (or failed to offer) them.

#### 3.1.1. Information and decision sharing

Some participants said they were allowed an active role in their support experience. This was viewed positively by many. Participant 1 reported that they could see their psychologist as regularly as they chose. The psychologist had passed this decision on to the young person, and appointments could be made as needed through a process of flexible and open contact.

*“He wouldn't say like, “oh, I’ll see you in two weeks, or I'll see you in a week” he'd say, “whenever you need to see me, you know the process”. Sometimes I'd go months without seeing him, and it'd be fine. But other times, I'd call up and say, “can I see you here at this time?” So yeah, I felt as though I was in control”. [Participant 1]*

Conversely, a lack of transparency and clarity was reported in some instances. An older participant felt that their age should have been considered when information about their course of therapy was provided. They felt that knowing more about their treatment would have allowed them to positively integrate this information into their journey. As such, their uncertainty meant that they needed to place a good deal of trust in the staff providing support.

*“I was a full-grown adult. So, I could have handled being told “you're receiving this type of therapy, because we think this will be beneficial to you” […] I think because I was in such a low place, I just willingly let myself walk into this building. And I had no idea what I could have been walking into”. [Participant 6]*

The following participant would have appreciated more information about the nature of their own mental health. Receiving a diagnosis was an important tool for helping them to understand their difficulties. It may also have helped them to feel valid in their help seeking: they felt that they were one of the only young people in their setting without a formal diagnosis.

*“I feel like they need to acknowledge that some people want a diagnosis. A lot of the time, people will go there, but they'll have a diagnosis. […] the nurse asked me why I was there, and I just I couldn't say anything, because no-one had told me that I had anything wrong”. [Participant 4]*

Despite the importance of transparency, other testimonies suggested that shared decision-making should be managed carefully. Professional insight should be appropriately applied to guide the process. Participant 5 felt that too much initiative was expected of them. They would have appreciated more help with identifying the most beneficial focus of their sessions.

*“It was put down to me to decide the focus of what we'd be talking about. But I think at the time, I didn't really know what I wanted to be talking about. Even though it was my choice, I think I chose the wrong thing […] I think I would have preferred to be told what to do a bit more and told what to focus on”. [Participant 5]*

Participant 6 actively hoped that decisions would be made on their behalf. They saw the commencement of professional help as an opportunity to pass on the onus of their difficulties. It should not, therefore, be assumed that high autonomy is universally valued. A considered balance should be drawn for each young person.

*“I think when I got to the point of needing therapy, it was like I was relinquishing my control, and I wanted someone else to do the work”. [Participant 6]*

**Corresponding self-assessment matrix principle(s):** Micro 1A, Micro 5B

**Level of agreement between staff and CYP testimony:** High

#### 3.1.2. Communication and continuity

Several participants' schools acted as a gateway to receiving mental health support. When support was not provided directly by their school, teachers were able to refer them to appropriate sources of support. This suggests that school staff have sufficient knowledge of a range of mental health services, and successful lines of communication exist between schools and these services. These qualities build a more seamless support acquisition process.

*“It came to me after speaking to multiple pastoral teachers. I have [mental health concern], and they said that they could help me by introducing me to people to talk to: mental health services”. [Participant 2]*

Participants who encountered multiple professionals and services across their mental health support experience said each new professional was equipped with at least a basic knowledge of them and of their mental health story. Participant 1 felt that when professionals asked them to elaborate on elements of their background, this was treated as part of the therapeutic process. This suggests an element of communication between providers, where gaps in professionals' knowledge are filled tactfully. This provides a smoother continuous care experience, that removes the strain of restating details about themselves to each person that they meet.

*“It’s always hard between telling, “are they asking me about me because they want to know my perspective on my life?” Or “they're asking me because they genuinely don't know?” But I think they had a general background of my life. And if they were asking me, it felt as though they were asking me for my perspective”. [Participant 1]*

The same participant spoke about becoming too old for NHS CAMHS support. Although they were successfully referred to an alternative source of support, they felt that the shift between NHS-based support, and this, was abrupt and difficult to navigate. A smoother post-18 transition would have improved the continuity of their care.

*“I wasn't perceived as high enough of a threat to be moved on to the NHS adult services. But my support worker at CAMHS referred me to a lower threshold thing […] which is really good […] I just think sort of a step-down service that could be used for anybody who had touch with CAMHS”. [Participant 1]*

**Corresponding self-assessment matrix principle(s):** Macro 2, Macro 5

**Level of agreement between staff and CYP testimony:** Moderate

#### 3.1.3. Needs-based support

Several participants felt that they were taken seriously, and that they were listened to. This allowed their support to be tailored appropriately to their individual needs and preferences.

*“Everything she said to me, everything I said to her, she took very seriously. And I really appreciated that she did that”. [Participant 3]*

This participant reported that their predicted duration of care was extended based upon their continued requirements. After their initial period of support ended, they were easily able to recommence at their own request, as their needs changed. The participant's decision to reengage with the support appears to have been aided by their previous positive experience, and the approachability of the staff they met.

*“I was supposed to [have support] for 6 weeks, but I think I had 8 or 9. Then a few months later, I asked to go back. There was nothing wrong with the treatment that I previously received. But the people there that I had; she was really nice. I asked to go back, so I did”. [Participant 4]*

Some participants, however, reported that their needs were not taken seriously enough. Participant 2 said that when they were younger, a member of their school pastoral team frequently raised irrelevant topics. This meant that they did not receive help with the issue with which they had originally been referred. Although it is unclear whether the pastoral worker lacked expertise in the appropriate area, or whether this experience represents a true example of poor listening, the resulting lack of needs-based support clearly impacted the participants’ desire to continue with the sessions.

*“I started seeing a school nurse, and the sessions were supposed to be about [mental health concern]. I explained to her, but she started talking to me about [an unrelated concern]. Every time I tried to draw away from the topic, she just kept on steering it back to that. It didn't last long after that, I just stopped seeing her”. [Participant 2]*

**Corresponding self-assessment matrix principle(s):** Meso 3B

**Level of agreement between staff and CYP testimony:** High

#### 3.1.4. Compassion and trust

This subtheme covers the personal qualities of support or care providers of mental health support that were memorable to the participants. Several participants described the professionals as kind people, who genuinely appeared to care about their wellbeing. The following participant describes that perceiving these qualities allowed a quicker development of trust.

*“It took a while to build up the trust to be able to speak to her […] and it only took a few weeks, because she came across as a very nice, genuine person to me”. [Participant 3]*

Trust is mentioned again in the following extract, where Participant 6's provider made them feel that nothing that they discussed would be passed on outside of the session. The participant detected clear signs that their provider had listened to them in previous sessions, which added a personalised element to the support they provided. This further developed the trust they felt. This professional was just one member of staff operating in a wider compassionate environment, that the participant sensed as soon as they entered the building for the first time.

*“I felt very much like all the things I was telling her were 100% confidential […] I felt very safe with her as my therapist. The way she would remember little details and always think of other ways I could have improved […] So, I think the actual genuine support that they gave young people, I could see that throughout the building”. [Participant 6]*

Participant 5 was impressed with the stoic attitude of their provider. The fact that they did not appear shocked or upset by the information that they disclosed contributed to a calmer and safer environment, where no topic was taboo.

*“She always had a friendly face on, even when I was telling her some really not nice stuff. She's very good at dealing with it in a way that I definitely couldn't if someone was telling me those kinds of things”. [Participant 5]*

### 3.2. The mental health journey

This theme covered participant experiences that related to the different stages of their personal mental health stories, and how these were accommodated by the services that they got support from.

#### 3.2.1. Beginnings

Several participants mentioned struggling with their mental health for a long time before they received support. Many referred to a specific moment, almost a “tipping point”, where they, a family member, or a teacher, realised that professional support was needed. They suggest that their mental health difficulties had built over time, developing from lower-level concerns that were not necessarily noticed by those close to them, to more severe challenges that greatly interfered with their functioning. Following feelings of depression from a young age, participant 4 spoke of one evening where they experienced a mental health crisis, and the emergency services were contacted.

*“I ended up becoming really depressed dead young […] I ended up calling 999. Because I just, I felt really bad one night […] I ended up having to go to hospital because I was a child”. [Participant 4]*

The participants talked about the various avenues through which their first contact with mental health support was accessed. Many were either referred to external support by their school, or received early support directly from their school. The next testimony describes the value of knowing that help is available. Even though participant 1 was not ready to engage with support when they were first approached, the process of opening dialogue by informing them of who they could turn to seemed important to them. Participant 1 was able to internally process the idea of receiving support, and they eventually approached the teacher on their own terms.

*“Whoever is on call at the time to deal with issues like this was like, “what's going on?”. I didn't speak to them. I was like, “none of your business”. A few weeks later, I approached this teacher and we sat down. We had a chat for about two hours, and I just cried and cried and cried”. [Participant 1]*

Participants valued building familiarity before their support formally began. This level of comfort made them feel more relaxed, and that any anxieties were at least partially ameliorated. This early breaking down of barriers between client and professional is likely to have enhanced the benefits of the support.

*“I was a lot more comfortable talking to her, and she knew some stuff about me as well. So, it was a lot more comfortable between both of us”. [Participant 2]*

For Participant 3, these early conversations were used to establish the nature of their needs, so that appropriate support could be given. Following this discussion, regular sessions were set up.

*“Before we started our sessions, I did meet with her. And she did ask me some questions just to get to know about my home life, my school life […] And then from there, she got a plan, because then she started saying that we'll meet up in these days”. [Participant 3]*

#### 3.2.2. Endings

When participants spoke about their support coming to an end, their level of preparedness was discussed frequently. The ending of support is a stressful time for many young people, and participant 3 stated that the topic was raised regularly in their sessions. This allowed them to imagine a time when the professional was not accessible, and to develop approaches to manage their concerns alone in the future.

*“She prepared me quite well. When she explained something to me, she would give me advice on how to remember things, and she'd say “don't forget that one day, I won't be here for you to come and speak to. So, you're gonna have to be able to cope on your own and have good strategies to deal with your mental health"”. [Participant 3]*

For some, the timing of their ending was less clear. Although the participant below appreciated that their professional decided over time how long their support would need to last, and that there were advantages as well as disadvantages to not knowing, they feel that their ending felt abrupt. Knowing earlier would have given them time to process the next stage of their support.

*“I just was randomly told one session, like, “Okay, this is your last of four sessions” or whatever. It was very surprising to me. If I'd known, I would have maybe seen it differently […] But I also just think that maybe making it more clear to me how I could have carried on receiving support if I needed to”. [Participant 6]*

Whilst many participants felt ready for their support to conclude when it did, some felt anxious and unsure. Participant 5 said that the topic of continuing need was not discussed thoroughly enough. They were left questioning whether they had made the most of their time, and the lack of conversation around this rendered them unsure of what further support they needed, or how to ask for it.

*“She told me that I can re-refer, and it'll probably be quicker than if I were to start again somewhere else […] But I do remember feeling very anxious about it ending like, “oh no, I don’t know if I've gotten everything out of this that I could have” […] the last session could have just been a bit longer, because I find it really difficult on the spot to know what I need […] it was just like, “okay, bye"”. [Participant 5]*

**Corresponding self-assessment matrix principle(s):** Micro 3A, Micro 3C, Micro 5A

**Level of agreement between staff and CYP testimony:** High

#### 3.2.3. Waiting

Several participants reported that whilst setting up regular support was not instantaneous after their initial referral, it was shorter than what they were told to expect. This suggests that professionals may give their clients larger timeframes to manage their expectations and avoid disappointment. The following participant waited a small proportion of the maximum duration that they were initially quoted. During this time, they were able to meet the professional to build familiarity before the official start.

*“It was actually pretty fast. When the pastoral teacher first put me down for it, she said there might be a long wait, like six to 12 weeks. Then I actually met [professional's name] before the sessions started. And then it was like three weeks after that. That's when I started seeing her”. [Participant 2]*

Participant 6 believed that their wait, to use a non-NHS service, negatively impacted their mental health. This may be a likely scenario for many, given that a high level of distress is often felt before support is initiated (see subtheme “beginnings”). Participant 6 spoke of the wide-spread issue of waiting times for mental health support, and how this has perhaps skewed perceptions of what an acceptable waiting time looks like.

*“It definitely was detrimental for me to have to wait three months. But in comparison, I know the NHS waiting list is insane […] But the three months, I think the fact that it was the minimum that she told me actually was really good. And I saw that as like, wow, amazing, that's so quick like, which is kind of messed up, I guess, that we think of three months as being quick”. [Participant 6]*

Another consequence of lengthy waits is that mental health concerns are not dealt with when they are the most salient. Participant 5 said that by the time their sessions began, although they still made use of the support, they had already come to terms with the difficulty they initially sought help for. During this time, in a worst-case scenario, where need is not professionally met, unhealthy coping strategies may be developed. These may be difficult to overcome if related difficulties re-emerge over time.

*“I signed up, and then it had been so long that I’d moved on from what I originally wanted to talk about. So, once I got there, I was like, maybe I'll use it because I am still struggling in other ways, but it definitely wasn't what I originally signed up to do it for”. [Participant 5]*

#### 3.2.4. Satisfaction with support

All participants stated that overall, they would recommend the type of support that they got to another young person. Participant 5 suggested that the broadness of their support means that they would suggest it to most people. They appeared to find the process insightful and enlightening, in that it helped them to identify the root causes of their difficulties.

*“The stuff that I was taught is very broadly applicable. The psychoeducation aspect of it, like “oh, this is where those symptoms are coming from” was really, really helpful. So yeah, I think that anyone… I say it all the time to my siblings, “go and get some cognitive behavioural therapy”. Can't recommend it enough”. [Participant 5]*

Some participants, although stating that their experiences were positive overall, would only recommend their support under certain circumstances. These participants discussed the nuances of their own support-seeking journeys, and said, therefore, that they could only truly endorse it to somebody who's circumstances were near-identical to their own. The following participant felt lucky to have received such good support, and they perceived their experience as the exception rather than the norm in terms of how smooth it was.

*“I felt so fortunate the entire time […] But that's just my experience. It's not what most people would say. I'd only be recommending the type I got, because I probably know, like six or seven people who've had a really, really bad experience with CAMHS. And it's a shame because I wouldn't want to recommend someone for them not to be getting good treatment”. [Participant 1]*

## 4. Discussion

This study explored the implementation of reformed CYP mental health service provision within the context of a recently devolved healthcare system. This broad aim was investigated through a variety of methodological lenses, to establish not only the improvements that have been made, but also the adequacy of the tools used to monitor this progress. Evaluating the plan that was set prior to the implementation of GM i-THRIVE, then cross-referencing professional and service user accounts of adherence to the THRIVE Framework's core principles, provided unique triangulated insights into an intervention across the entirety of its implementation timeline.

Table 2 showed that most criteria of the QIT were evidenced within GM i-THRIVE's implementation plan, and the evaluative self-assessment matrix. Most criteria that were either not evidenced, or ambiguously evidenced, fell under the remit of the implementation plan (QIT stages 1–5) rather than the self-assessment matrix (QIT stage 6). Whilst this could suggest that the plan was less sufficient than the matrix, we are reluctant to assert that these processes were, without question, not undertaken in GM i-THRIVE's implementation process. It is plausible that certain elements of the QIT were not deemed relevant enough to feature within GM i-THRIVE's plan and matrix. For example, Point 4.5 of the QIT, “work with technical assistance providers to implement the innovation” has limited relevance to GM i-THRIVE, given the programme's broader focus. Additionally, whilst most QIT points were straightforward to cross-evaluate, those components classified as “unclear” may simply be worded differently depending on an intervention's nuances. This can make it difficult to ascertain a clear match. For example, point 2.4 of the QIT “establish practices that counterbalance stakeholder resistance to change” was not explicitly referred to within the implementation plan, however cross-sector approval was mentioned. This suggests that a level of commitment to GM i-THRIVE was sought before proceeding. In response to the first research question of this study, we conclude that GM i-THRIVE was equipped with a suitable foundation prior to implementation, and with a strong method of evaluating progress during the implementation process. The remaining findings should, therefore, be considered in the context of these bases.

Figure 3 shows that progress across Greater Manchester, although not linear in every instance, was made between 2018 and 2021 on all nine self-assessment matrix principles included in the analysis. A gradual shift towards THRIVE-aligned practice is broadly evident. Each principle will be discussed in the context of the reported experiences of CYP who received support within this time frame, but before this, it is worth noting that not all subthemes could be appropriately compared to a matrix principle. This element of conceptual mismatch relates to the fact that an inductive approach was taken—hence, the qualitative data were not forced into deductive codes that related to the principles. Similarly, the topic of one principle, Micro Principle 2, “people (staff, CYP and families) are clear about which needs-based group they are working within for any one person at any one time, and this is explicit to all” did not feature in the interview data. This is perhaps indicative of a limitation that can be applied to all meta-inferences that we will draw within this section: that evidence of THRIVE principles in CYP testimonies can only be inferred. They are not likely to use or even know the exact terminology used in the Framework, especially if this complex language is not consistently used by professionals in their interactions with CYP. Along with the other principles, Micro Principle 2 showed improvement over the implementation period, adherence to it was rated as relatively low (Figure 3), which may reflect this. Agreement between the interview data and the staff accounts in Figure 3 was generally high. Half of the generated themes matched at least one matrix principle. This suggests that the self-assessment matrix is a relatively accurate reflection of the care experiences of CYP in Greater Manchester, and also that CYP can provide relevant and accurate accounts of this support (13, 38). These substantiations will now be explored in turn.

The interview data were split into two overarching themes (see Figure 4). The first of these, “qualities of the service”, covered four subthemes. “Information and decision sharing” explored the topic of control and taking an active role in the support process. Agreement between staff self-assessments and the points raised in this subtheme was deemed good. By 2021, staff reported their incorporation of shared decision-making moderately, yet with a clear improvement since 2018. This substantiates participants' mixed reports on their perceived ability and desire for involvement. Clinicians' communication skills, understandable information, and CYP capacity were just three factors identified as important for shared decision-making in CYP mental health in previous research (39). This subtheme supports the finding that even when a young person's mental health does not allow them to be fully involved in decision-making, open communication and the transparent presentation of information should still be offered, as deemed appropriate through the listening process (39).

Professional agreement with points raised in the subtheme “communication and continuity” was considered moderate. The self-assessment principles relating to multi-agency involvement, and integrated care, were consistently rated highly, and CYP generally reported a well-connected experience. However, the difficulties with the transition between child and adult mental health services were raised.., This is, unfortunately, a common source of distress, especially as these changes in care coincide with a number of other life transitions in late adolescence (40, 41). Disrupted support can result in feelings of stress, struggles with coping, and an increased burden on family members to provide support (42). However, a transition that is well-planned, gradual, and needs-based is more likely to be experienced positively (40).

In the next subtheme “needs-based support”, CYP responses were mixed in terms of whether they felt that their requirements were noticed and actioned.. These experiences matched closely with professionals' scoring of meso principle 3B which relates to needs-based care: a principle that was rated moderately, but with a steady improvement year on year. Feeling listened to is one of the most valued aspects of support for CYP, with professional understanding key to having mental health needs met (43). The final subtheme within the “qualities of the service” theme, “compassion and trust” did not relate directly to a matrix principle. However, participants readily reported on the kindness of the professionals they met. Feeling that professionals genuinely care for their wellbeing contributes to a positive support experience (43), where better outcomes may be more likely (44).

The second theme, “the mental health journey”, also comprised four subthemes. In “beginnings”, participants valued building familiarity to foster trust prior to an official start—a process which should not be rushed (45). This subtheme emphasises the importance of early recognition of mental health difficulties in CYP, so that support can be given before they exacerbate. Mental health promotion programmes, such as those offered in schools, can help CYP to identify concerns (46), reduce stigma (47), and increase help-seeking tendencies (48).

The subtheme “endings” saw some participants feeling well-prepared for their support coming to an end, whereas for others, the ending felt abrupt. Agreement with staff reports was good.Although staff recognised endings important parts of the therapeutic journey, and that discussions of timeframes were often had, they felt that the limitations of help were not always made clear to CYP. The honest setting of expectations and defining of outcomes at the outset of support is vital (49). Itis therefore important that GM i-THRIVE continue to emphasise the importance of such discussions across the sector.

In the subtheme of “waiting”, participants felt that their expectations were managed well, but only within the wider context of the normalisation of long waits. A detrimental impact of long waits on mental health was reported. Other qualitative studies have reported similar negative consequences of long waits. This is a well-documented issue within CAMHS as well as the wider NHS (9, 11), with exacerbation of concerns frequently reported as a consequence of delays (50). The subtheme was titled “satisfaction with support”. Whilst participants were keen to recommend their support, however this endorsement occasionally came with the caveat that their good experience was an isolated incident. Continuing to monitor CYP and parent satisfaction with support (51) will be vital during GM i-THRIVE's embedding phase.

### 4.1. Strengths, limitations, and future directions

The key strength of the present study is the mixed-methods approach, which sought to seek consensus across a range of sources, from multiple informants. Greene et al. (29) stated that a typical way of mixing quantitative and qualitative approaches is to use the former to assess empirical outcomes of a programme, whilst using qualitative testimonies to gauge how well these outcomes have been implemented. Our approach echoed this, by looking first to the quality of guidance documents and measures, before examining how GM i-THRIVE's outcomes were measured by staff, then finally asking recipients of the intervention how their experiences reflected THRIVE-aligned support. The simultaneous bidirectional approach (36) taken meant that the three research questions were all considered within the context of one another to draw the study's final conclusions. This combines the strengths of corroborating testimonies from multiple informants, which is important for both implementation evaluation (52), and studies on CYP mental health services (22, 23, 53).

However, despite this methodologically strong approach, our findings must be considered within the context of their limitations. First, the overwhelmingly inductive qualitative approach led to an imperfect cross-over between the subthemes and the principles of the matrix presented in Figure 3. This meant that a true comparison of staff and CYP accounts was difficult to make in some areas. However, the subthemes and their corresponding extracts were used to emphasise the overarching thematic points that were made, and we believe that adding an inductive element to this analysis provided a more genuine representation of the experiences of our participants. The approach allowed us to incorporate ideas into the analysis that, had the participants not raised them, we would not necessarily have thought of. This, overall, led to a stronger and more representative evaluation.

An inductive approach was especially important given the study's small sample of CYP, a result of the challenging nature of recruitment, to ensure that the interviews were themed as suitably as possible. Similar studies undertaken in the health research field (54), or with a niche set of inclusion criteria (55) have used similar-sized samples, and some researchers have indeed reached thematic saturation with a small number of participants (56). Some even suggest that a smaller sample can lead to deeper qualitative enquiry (57). However, our small sample might imply that we were not able to harness a wide range of experiences with mental health support. This is a plausible limitation given that the included testimonies were overwhelmingly positive. This suggests that those who had very negative experiences were not identified as potential participants, perhaps because factors associated with the support provider (58) and the therapeutic relationship (59) are linked to drop out or disengagement with services. Focussed efforts on reaching these CYP would have diversified the range of views captured, and we recommend that future evaluation of GM i-THRIVE attempts to make this effort. A similar point can be made regarding the homogeneity of the sample, given that all participants were either in the final stages of receiving support, or they had already been discharged. This has undoubtedly restricted the variety of experiences expressed. However, whilst it would have been useful to capture the opinions of those in the middle of their support journey, or who were on a waiting list to receive support, the ethical considerations surrounding approaching and interviewing CYP who are potentially at a very vulnerable stage led us to eliminate these groups from our recruitment pool.

The reporting of predominantly positive views may also explain the agreement between CYP and professionals on THRIVE alignment, especially given that other studies comparing accounts from both have not found such close consensus (23). As all participants were aged 13 or over, whether the findings can be applied to the experiences of younger children is uncertain. As this age parameter was set to ensure that participants were capable of engaging fully by providing sufficiently detailed accounts, the support experiences of younger children may need to be accessed through their parents or carers, even though this approach accompanies its own set of limitations relating to the salience of reported outcomes (22). In further relation to transferability, we would recommend that other regions within England who are in the process of aligning their CYP mental health provision to the THRIVE Framework conduct their own qualitative studies with CYP, to corroborate with professional accounts of progress. Unique considerations associated with the North of England, an area with poorer deprivation-associated mental health than the South (60–62), may not be applicable to other regions in the country.

The final limitation that we wish to raise is that although the data within this study covered the entire four-year initial implementation period, it should still be treated as a cross-sectional account. Within GM i-THRIVE, evaluative work should continue, including further conversations with CYP. This is because implementation should not be assumed a linear process. Numerous influencing factors, both wider and organisational, continuously influence progress and sustainability (63). Additional monitoring is especially necessary following the COVID-19 pandemic and its emerging impact on CYP mental health (64) and the provision of mental health services (34).

### 4.2. Conclusions

Here, we summarise the meta-inferences made by combining the lines of enquiry in this mixed-methods study. GM i-THRIVE's initial plan set a solid foundation for the implementation work that was to follow between 2018 and 2021, and the embedding period that will follow this. Similarly, the self-assessment matrix was a suitable tool with which to assess alignment of services to the THRIVE Framework. Under this context of good quality planning and measurement, progress was made towards aligning services to the THRIVE Framework. Although limitations were identified, professional staff working within these services, and the CYP receiving support and care, tended to agree on what mental health provision looked like during the reform period. Given the rich insights offered by the study's participants, we recommend continued discourse with service-users with a range of support experiences as the intervention continues to be embedded.

The triangulation of methods in the present study aimed to deliver a practical and original insight into how implementation science feeds down to those in receipt of an intervention. The comparison between the unique experiences of CYP, and the opinions of progress expressed by those implementing the programme, provides valuable understanding of whether implementation and evaluation tools, in isolation, can produce accurate and valid representations that are reflected in the experiences of those in receipt of care. The study produces helpful findings that can be used to guide the future of GM i-THRIVE, in addition to providing a valuable and unique contribution to mixed-methods research, particularly that which pertains to implementation evaluation.

## Data Availability

The datasets presented in this article are not readily available because of privacy concerns owing to potentially identifiable information within the interview extracts. This is in accordance with the research governance policy of the University of Manchester. However, the data may be available from the corresponding author (EB) on reasonable request. Requests to access the datasets should be directed to emily.banwell@manchester.ac.uk.
