# Supplementary material for: Reformed child and adolescent mental health services in a devolved healthcare system: a mixed-methods case study of an implementation site
Source: Front Health Serv. 2023 May 5;3:1112544. doi: 10.3389/frhs.2023.1112544 (PMC10196272; doi:10.3389/frhs.2023.1112544)
Supplement: Supplementary file 1 [file Datasheet1.docx]

| **Point** | **i-THRIVE Approach to Implementation Phase 0: Set Up** |  |
| --- | --- | --- |
| 0.1 | Establish cross sector approval to proceed with i-THRIVE |  |
| 0.2 | Have a named lead for implementing THRIVE |  |
| 0.3 | Set up multi-agency Programme Board (include senior leadership from CCG, health provider(s), local authority, education, third sector) |  |
| 0.4 | Undertake stakeholder mapping |  |
| 0.5 | Create communications and engagement plan based on stakeholder analysis |  |
| 0.6 | Multi-agency working group established |  |
| 0.7 | Site leads/sponsors identified (where i-THRIVE delivered across multiple sites) |  |
| 0.8 | Establish communications functions (contact databases, shared folders, website) |  |
| 0.9 | Create high level project plan |  |
| 0.10 | High level project plan approved by Programme Board |  |
|  |  |  |
| **1.0** | **i-THRIVE Approach to Implementation Phase 1: Engagement, Understanding and Planning** |  |
| 1.1 | Key messaging for i-THRIVE project established - goals, aspirations, local context |  |
| 1.2 | Whole system stakeholder engagement event |  |
| 1.3 | Pathway mapping workshop |  |
| 1.4 | Staff consultation |  |
| 1.5 | Young people and families consultation |  |
| 1.6 | Review Quantitative data and fill gaps |  |
| 1.7 | Qualitative review of feedback collected |  |
| 1.8 | Service performance review (including population need, demand, flow, experience of service, participation levels, clinical outcomes, efficiency, current shared decision making practice etc) |  |
| 1.9 | Mapping of current outcome measures used across health, social care, education and voluntary sector |  |
| 1.10 | Understanding your data workshop |  |
| 1.11 | THRIVE Assessment Tool workshop |  |
| 1.12 | Engagement with wider system re THRIVE-like baseline score |  |
| 1.13 | Prioritisation and gap analysis workshop |  |
| 1.14 | Redesign workshop |  |
| 1.15 | Creation of local model for i-THRIVE |  |
| 1.16 | Finalise implementation plan |  |
| **2.0** | **i-THRIVE Approach to Implementation Phase 2: Building Capacity** |  |
| 2.1 | Review of staff skills for THRIVE-like working |  |
| 2.2 | Review of staff capacity for delivery of new model |  |
| 2.3 | Plan for recruiting to deliver new model |  |
| 2.4 | Review of training opportunities available across all organisations |  |
| 2.5 | Creation of workforce development plan |  |
| 2.6 | Development of new commissioning specification |  |
| 2.7 | Identification and creation of local champions and implementation leads |  |
| 2.8 | Training for implementation leads (knowledge of change management, i-THRIVE Approach to Implementation and QI) |  |
| 2.9 | i-THRIVE Academy training for multi-agency front line staff and managers |  |
|  | - Shared decision making |  |
|  | - Getting Advice and Signposting |  |
|  | - When to stop treatment |  |
|  | - Risk Support |  |
|  | - i-THRIVE Grids |  |
|  | ACE / Trauma training |  |
| 2.10 | Multi-agency workshop event to reflect on learnings from i-THRIVE Academy and other training |  |
| 2.11 | Delivery of other training and development as set out in workforce development plan |  |
| 2.12 | Review of workforce development delivery and plans for ongoing work |  |
| **3.0** | **i-THRIVE Approach to Implementation Phase 3: Implementation** |  |
| 3.1 | Ongoing review of implementation plan agreed at end of phase one |  |
| 3.2 | Detailed implementation planning finalised with lead for each project identified |  |
| 3.3 | Delivery of implementation plan as specified *import detailed implementation plan into this section* ***The implementation plan should include - as a minimum - the following elements which are required in order for a site to be assessed as 'THRIVE-like' in line with the THRIVE Assessment Tool:*** |  |
|  | > Children and young people's mental health is included in the LTP and the STP and implementation plan is in place for delivering outcomes |  |
|  | > Multi-agency strategy and policy for delivering improved outcomes for children and young people's mental health operational with clear and measureable goals |  |
|  | > Multi-agency commissioning board operational with joint accountability for delivering strategy |  |
|  | > Shared outcome framework for the multi-agency commissioning board is in place |  |
|  | > Annual commissioning cycle (multi-agency) operational supported by formal mechanism for service performance, outcome and preference data to inform the process |  |
|  | > Joint budgets to support delivery of strategy established with processes in place across all involved agencies |  |
|  | > Protocols established for regular sharing of performance and outcomes data across all agencies and commissioners |  |
|  | > Introduction of systematic preference data collection across all agencies and sectors |  |
|  | > All QI projects are undertaken using recognised methodology |  |
|  | > Outcomes and process measures are collected routinely and this data is used to help shape service provision, manage performance and deliver interventions **full list of measures is available from i-THRIVE programme team |  |
|  | > Local pathways structured to deliver care according to five THRIVE needs based groups |  |
|  | > Multi-agency assessment that categorises children and young people into the THRIVE needs based groups is operational |  |
|  | > THRIVE needs based group recorded for all cases |  |
|  | > Implementation of i-THRIVE Grids to support shared decision making |  |
|  | > Record of shared decision making documented for all cases |  |
|  | > Population health and wellbeing offer available to all children and young people |  |
|  | > Targeted prevention and resilience building offer available to children and young people who we know are more likely to require support with their mental health and wellbeing |  |
|  | > Digital front end is available for children, young people and their families |  |
|  | > There is a self-help and peer support offer available to children, young people and families |  |
|  | > Senior mental health practitioners (band 8 or above) involved in all advice and signposting |  |
|  | > Digitally enabled database of full range of community providers is operational |  |
|  | > In reach from CAMHS to schools is operational |  |
|  | > In reach from CAMHS to primary care is operational |  |
|  | > CAMHS outreach to hard to reach groups is operational |  |
|  | > Evidence of families being involved in development of care plans in all cases |  |
|  | > Evidence of therapy being aligned with NICE guidance for all cases |  |
|  | > Recorded note of treatment goals and expected outcomes for all cases |  |
|  | > Children, young people and their families are managed within the recommended number of therapy sessions |  |
|  | > Reasons for ending proforma completed for all cases with treatment |  |
|  | > Multi-agency structures and protocols for providing risk support |  |
|  | > Multi-agency THRIVE plans documented for all risk support cases |  |
| 3.4 | Year one of implementation (details to be added) |  |
| 3.5 | Year one progress review and refine plan for year two |  |
| 3.6 | Year two of implementation (details to be added) |  |
| 3.7 | Year two progress review and refine plan for year three |  |
| 3.8 | Year three of implementation (details to be added) |  |
| 3.9 | Year three progress review and refine plan for year four |  |
| 3.10 | * Continue as necessary in yearly cycles* |  |
|  |  |  |
| **4.0** | **i-THRIVE Approach to Implementation Phase 4: Embedding and Sustaining** |  |
| 4.1 | Establish learning collaborative |  |
| 4.2 | Learning collaborative |  |
| 4.3 | Embedding and sustaining year one implementation projects |  |
| 4.4 | Embedding and sustaining year two implementation projects |  |
| 4.5 | Embedding and sustaining year three implementation projects |  |
|  |  |  |
|  |  |  |
|  |  |  |
|  |  |  |
|  |  |  |
|  |  |  |
|  |  |  |
|  |  |  |
|  |  |  |
|  |  |  |
|  |  |  |
|  |  |  |
|  |  |  |
|  |  |  |
|  |  |  |
|  |  |  |
|  |  |  |
|  |  |  |
|  |  |  |
|  |  |  |
|  |  |  |
|  |  |  |
