# Supplementary material for: Reformed child and adolescent mental health services in a devolved healthcare system: a mixed-methods case study of an implementation site
Source: Front Health Serv. 2023 May 5;3:1112544. doi: 10.3389/frhs.2023.1112544 (PMC10196272; doi:10.3389/frhs.2023.1112544)
Supplement: Supplementary file 2 [file Datasheet2.docx]

*Introduction:*

Hi [name], how are you /weather/have you come out of school/college to have this interview/what do you have planned for the rest of your day…

Thank you for coming along to do this interview with me today. I would just like to ask you a few questions about your experiences with getting help from [CAMHS/HYMS/other]. If you don’t want to answer any of the questions, that’s no problem at all. Please just say, and then we can skip it. We can also stop the interview at any time, there is absolutely no pressure, so just let me know. Also, please shout up if something I ask doesn’t make sense, or you don’t understand a question. Everything that you say today is completely private and confidential, unless I become worried that any harm or danger will come to you, then I would need to speak to [gatekeeper name] about this. Apart from this, nothing at all will be told to anyone outside of this call. I will use Zoom’s voice recorder to record your interview, so that I can listen to it and type it up, but I won’t use your name at all during this recording, so that what you say can’t be matched to you. As I said before, we can stop at any time you wish, and please do interrupt at any time if you have any questions. I will turn the recorder on now…

CYP:

1. So, to begin, it would be great if you could tell me a little about yourself, and how you came to need a bit of help with your mental health?
2. Which services have you got help from in the past?

*Prompts:*

Did you go to CAMHS appointments? Did you have any help at school on top of this?

1. For how long was this? You can give a rough guess if you can’t remember exactly!
2. I’d like to know what it was like when you first started receiving help at [xxx].
3. If you can remember back to your earliest appointments, do you feel like you were allowed to make some of your own choices about the help you got?

*Prompts:*

Did you have the option to tell the staff what times or places were good, or not so good, for you to attend?

Were you allowed to choose whether you had sessions on your own, or in groups?

If you said you didn’t like the idea of a certain thing, how much do you feel like they listened to you? What other options did they give you?

*If gives positive answers:*

Could you tell me how being able to make some of these choices made a difference to you? Prompt: Did it help much?

*If gives negative answers:*

If you had been given a bit more freedom to get involved in these decisions, how do you think this would have changed things for you?

1. Thinking back to the beginning, were you happy with the length of time you waited in between any of the stages of, for example, first being referred by your doctor or your school, to when you managed to get an assessment? Or between getting an assessment and starting treatment?

*Prompt:*

Do you think it took a long time, or was it quite quick?

Can you remember roughly how long it was you waited to [get referred/get assessed/start treatment]?

1. If you saw any new people during your time, to what level did you feel that they knew about you before seeing them?

*Prompt:* Did you ever have to repeat your story to several people, or tell them why you were there? Or do you think they knew, or remembered, a little bit about you before each session?

1. I would now like to hear about the time when your sessions finally came to an end.
2. Why did your sessions end? (Age, i.e. transfer to adult services, improvement, etc.)
3. When they did finally stop, did you feel ready for this?

If yes: Could you tell me a little bit about what they did to prepare you for them ending? Prompts: For example, did your sessions become more spaced out, happening less regularly?

If no: Has any alternative help been put in place for you?

1. Who was involved in the decision for you to stop attending?
2. Do you think anything could have been done to make the end of your time feel smoother?
3. To what level do you think you were listened to at this time?
4. Thinking right the way across your time with the services, what do you think could have made the help better, or more suitable, for you?
5. Overall, did you feel like your thoughts and opinions were listened to?

*Prompts,* if doesn’t expand: Could you tell me about a time during your sessions where you felt like your feelings were taken into consideration?/Could you tell me about a time where you think they could have listened to you a bit better?

1. Would you recommend getting this type of help, to another young person who might need it? What do you think could be improved to make it better for them?
2. Ask depending on whether they have offered up info/been proactive with answering before:

Does anything else come to mind that you were really impressed with during your time, or on the other hand, was there anything that you found really frustrating? It would be interesting for me to hear about it.

Debrief:

Participant will be thanked for their participation, and given a further opportunity to ask questions.
