# Supplementary material for: Reformed child and adolescent mental health services in a devolved healthcare system: a mixed-methods case study of an implementation site
Source: Front Health Serv. 2023 May 5;3:1112544. doi: 10.3389/frhs.2023.1112544 (PMC10196272; doi:10.3389/frhs.2023.1112544)
Supplement: Supplementary file 3 [file Datasheet3.docx]

Line graph visualisations of Greater Manchester’s self-reported adherence to GM i-THRIVE’s core principles from 2018-2021.
